# Improvement in Diagnosis and Management of Nosocomial Pneumonias in a Cardiovascular Surgery Intensive Care Unit: A Multidisciplinary Approach

**DOI:** 10.3390/antibiotics13070590

**Published:** 2024-06-26

**Authors:** Kirstin J. Kooda, Alejandra A. Zambrano, Dylan L. Kosaski, Leah Higbe, William Brian B. Beam, J. Kyle K. Bohman, Erica D. Wittwer, Steven D. Brady, Allison M. LeMahieu, Madiha Fida, Aditya Shah

**Affiliations:** 1 Department of Pharmacy Services, Mayo Clinic, Rochester, MN 55902, USA; kosaski.dylan@mayo.edu; 2 Department of Infection Prevention and Control, Mayo Clinic, Rochester, MN 55902, USAhigbe.leah@mayo.edu (L.H.); 3 Department of Anesthesiology, Mayo Clinic, Rochester, MN 55902, USA; 4 Department of Laboratory Medicine and Pathology, Mayo Clinic, Rochester, MN 55902, USA; 5 Department of Quantitative Health Sciences, Mayo Clinic, Rochester, MN 55902, USA; 6 Division of Public Health, Infectious Diseases and Occupational Medicine, Mayo Clinic, Rochester, MN 55902, USAshah.aditya@mayo.edu (A.S.)

**Keywords:** nosocomial pneumonia, informatics, visual analytics, antimicrobial stewardship, diagnostic stewardship, infection prevention and control

## Abstract

Background: While criteria for the diagnosis of nosocomial pneumonias exist, objective definitions are a challenge and there is no gold standard for diagnosis. We analyzed the impact of the implementation of a logical, consensus-based diagnostic and treatment protocol for managing nosocomial pneumonias in the cardiovascular surgery intensive care unit (CVS-ICU). Methods: We conducted a quasi-experimental, interrupted time series analysis to evaluate the impact of a diagnostic and treatment protocol for nosocomial pneumonias in the CVS-ICU. Impacts were measured relative to patient outcomes, diagnostic processes, and antimicrobial stewardship improvement. Descriptive statistics were used to analyze results. Results: Overall, 35 pre-protocol and 39 post-protocol patients were included. Primary clinical variables suggesting pneumonia in pre- and post-protocol patients were new lung consolidation (50% vs. 71%), new leukocytosis (59% vs. 64%), and positive culture (32% vs. 55%). Appropriate diagnostic testing improved (23% vs. 54%, *p* = 0.008) after protocol implementation. The proportion of patients meeting the criteria for nosocomial pneumonia (77% vs. 87%) was not statistically significant, though more patients in the post-protocol group met probable diagnostic criteria (51% vs. 77%). Duration of therapy was not significantly different (6 days [IQR = 5.0, 10.0] vs. 7 days [IQR = 6.0, 9.0]). Conclusions: The implementation of a diagnostic and treatment protocol for management of nosocomial pneumonias in the CVS-ICU resulted in improved diagnostic accuracy, advanced antimicrobial and diagnostic stewardship efforts, and laboratory cost savings without an adverse impact on patient-centered outcomes.

## 1. Introduction

The lack of gold standard diagnostic testing for hospital-acquired pneumonia (HAP) and ventilator-associated pneumonia (VAP) often leads to overdiagnosis, unnecessary testing, and overtreatment, with a significant downstream impact on infection prevention and control and antimicrobial stewardship by potentially fostering multidrug resistance, resulting in increased costs [1,2]. Although there are specific criteria for HAP and VAP diagnosis, objective definitions are challenging, and various subjective criteria must often be considered [3,4]. The existing literature demonstrates that physicians consistently overestimate the diagnostic probability of VAP in the ICU [5,6].

In addition to the general ICU challenges, patients undergoing cardiovascular surgery (CVS) are medically frail and have multiple comorbidities [7]. Surgical procedures that these patients undergo could include valvular repair or replacement, coronary artery bypass surgery, aortic root replacements, and other invasive procedures [7]. Owing to extensive CVS, their cardiac function is seldom normal. As a result, several of these patients have inflammatory syndromes after surgery that include a cascade of leukocytosis, fragile respiratory status, and hemodynamic shifts requiring vasopressor support [8,9]. These confounding symptoms may lead care teams to suspect a clinical case of HAP/VAP, particularly when the consequences of missed infection and delayed antibiotic treatment in the critically ill are well established.

HAP and VAP add a significant amount of morbidity, mortality, and health care expenditure per episode, particularly in the CVS population [1,10]. Furthermore, these are patients particularly vulnerable to adverse effects of antimicrobial overexposure, including infections like *Clostridium difficile* and issues with the promotion of multidrug-resistant organisms, and optimization of both diagnosis and duration of treatment is necessary to minimize these risks [11,12,13].

This study aims to propose a logical, consensus-based diagnostic and treatment protocol for managing HAP and VAP in the cardiovascular surgery intensive care unit (CVS-ICU) and evaluate its impact on patient outcomes, diagnostic processes, and antimicrobial stewardship.

## 2. Methods

**Figure 1:**
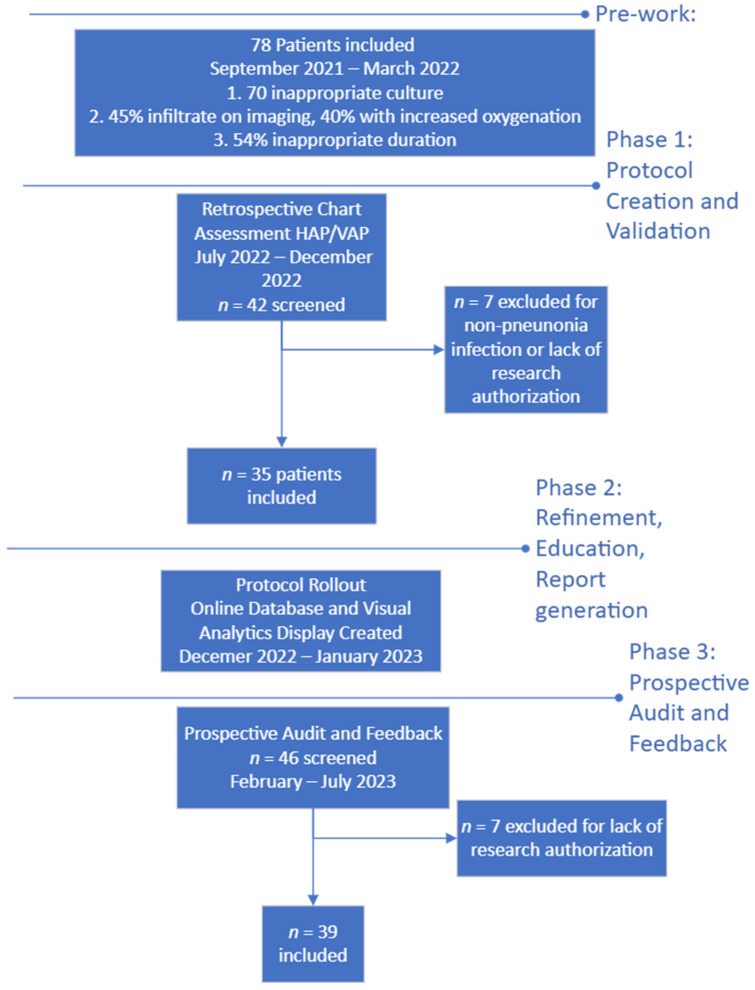
Flow diagram of protocol development and timeline.

In this quasi-experimental, interrupted time series analysis, we created, validated, and implemented a diagnostic and treatment protocol for HAP and VAP and evaluated the impact of it in immunocompetent patients admitted to the CVS-ICU (Figure 1).

This project was approved by the institutional review board (IRB).

Project design:

### 2.1. Pre-Work

Data obtained during this phase of retrospective clinical chart review (variables collected listed in Table 1) demonstrated three primary issues, as follows.

1. Patients who were not immunocompromised hosts (ICHs) were undergoing culture evaluation targeting ICH pathogens. Instead of just Gram staining and bacterial cultures of respiratory samples, orders included fungal and mycobacterial smears and cultures, which represents a significant burden on the microbiology lab. Each fungal culture is held for 24 days, and each mycobacterial culture is held for 42 days. We discovered that the order set used for processing bronchial alveolar lavage (BAL) samples automatically included fungal and mycobacterial smears and cultures. This led to frequent isolation of airway colonizers (e.g., *Candida* spp.) that would not typically need antimicrobial treatment, and no patient was found to have positive mycobacterial results [14]. Virologic respiratory panels were intermittently sent, and no patient received targeted antiviral therapy.
2. Patients were given broad-spectrum antibiotics (coverage for methicillin-resistant *Staphylococcus* aureus and *Pseudomonas aeruginosa*, including vancomycin, cefepime, meropenem, and piperacillin–tazobactam) for an average of 7 days in culture-negative and 9.3 days in culture-positive patients, even without data necessitating broad therapy.
3. Patients were given full courses of antibiotic treatment (e.g., vancomycin, cefepime, meropenem, and piperacillin–tazobactam) despite not meeting clinical criteria for pneumonia.

### 2.2. Protocol Creation and Validation

After assessment of the relative likelihood of pneumonia amongst the patients prescribed antibiotics, stakeholders from the CVS-ICU, infectious disease, and infection prevention and control (IPAC) were gathered to evaluate potential improvements that could be made in the diagnosis and treatment of these syndromes.

**Figure 2:**
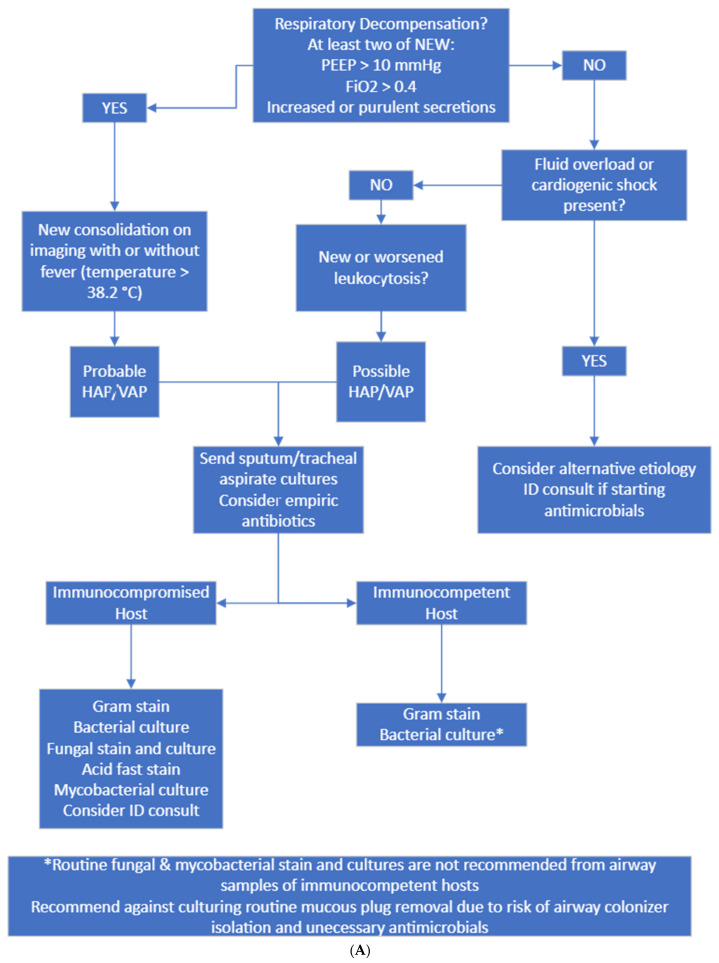

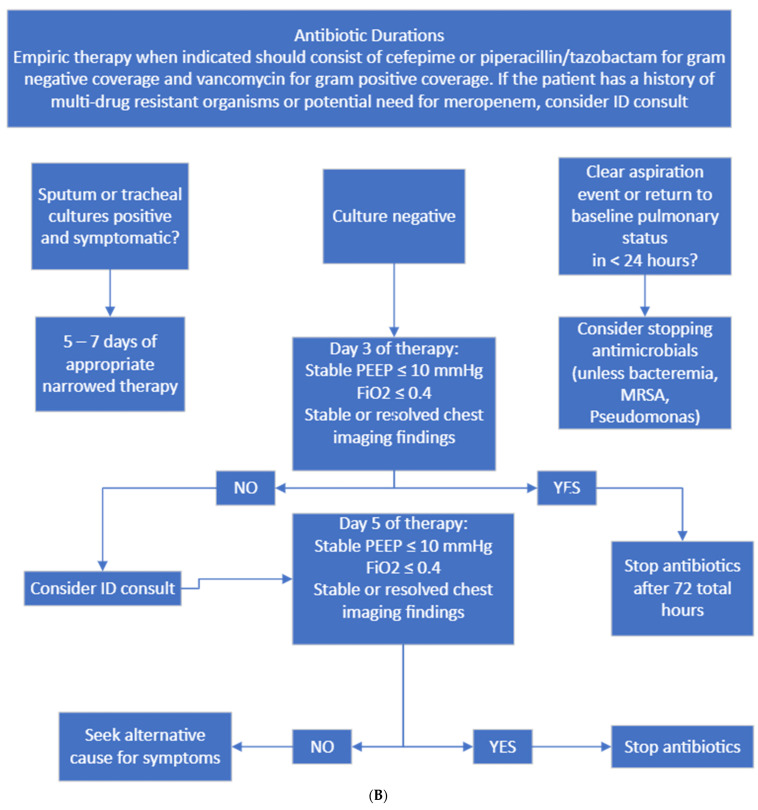
(**A**) Diagnostic and culture algorithm. (**B**) Antibiotic selection and duration.

The stakeholders created an agreed-upon set of criteria for diagnostic accuracy, culture attainment (Figure 2A), and treatment regimens and durations for HAP/VAP (Figure 2B). A multidisciplinary group of CVS, critical care, anesthesia, infectious diseases, IPAC, and antimicrobial stewardship clinicians apart from the initial stakeholders were consulted, and the protocol was modified based on their input. These included physicians, nurses, pharmacists, and advanced practice providers. The final recommendations were collated from multiple sources, including guidelines and primary literature [2,15]. The proposed protocol was retrospectively assessed during July–December 2022. Patients eligible for inclusion were similar to the preliminary phase, including prescription of antibiotics with an indication of respiratory tract infection, not admitted for immediate post-heart or -lung transplant care, and not experiencing another infection source. The primary goal was assessing diagnostic accuracy and treatment accuracy.

Probable HAP/VAP was considered when patients had new respiratory decompensation with new consolidation on imaging with or without fever. Possible HAP/VAP was considered when patients did not have new respiratory decompensation but had a new leukocytosis. Unlikely HAP/VAP was considered when patients met no diagnostic criteria. Appropriate diagnostic testing was only Gram staining and bacterial culture sent on respiratory samples from immunocompetent hosts and Gram staining, bacterial culture, fungal staining and culture, and mycobacterial staining and culture on immunocompromised hosts. Immunocompromised hosts were those who had a distant history of organ transplantation, chemotherapy or monoclonal antibody exposure or steroid dose > 20 mg prednisone equivalent daily for >14 days. Appropriate empiric antibiotics were vancomycin plus cefepime or piperacillin–tazobactam. If patients had a known history of multidrug-resistant organism infection, meropenem was considered appropriate.

To resolve the issue of excessive fungal and mycobacterial testing on culture, we modified the laboratory order set to no longer auto-select fungal and mycobacterial cultures and added the immunocompromised host diagnostic pathway on our protocol.

An automated report was created from the electronic health record (EHR) to virtually identify patients in the CVS-ICU with antibiotics prescribed for an indication of pneumonia. This report was reviewed daily, and prospective chart review occurred to collect data used to manually assign protocol adherence for both the diagnostic protocol (Figure 2A) and treatment protocol (Figure 2B). In addition to the manual assignment of protocol adherence by the clinicians, the team developed rule-based logical automation to assign adherence and non-adherence for both the diagnostic and treatment protocols. In cases of non-adherence, the automation would also assign reasons for non-compliance based on the criteria collected through chart review documented by the clinicians. The automated determination was refined and validated with chart review from AS, KK, AZ, and LH. Furthermore, the data collected to display adherence rates for the protocols were collated using data visualization software that allowed for real-time metric sharing with CVS-ICU providers regarding their performance for each section of protocol adherence.

Clinical information collected regarding the patients included basic demographic information, laboratory and physiologic data, and outcome data including duration of antibiotics, ICU and hospital length of stay, and duration of mechanical ventilation.

The primary hesitation from ICU providers related to a perception that they would not be permitted to initiate antibiotics if they had clinical concern for HAP or VAP. Group consensus was attained regarding short-course antibiotics in the case of negative cultures and symptom resolution. Multi-level provider dissemination and education on the protocol was completed during December 2022, and a one-month delay period was used to allow for natural provider behaviors to resume without the influence of education.

### 2.3. Protocol Implementation and Data Collection

After the period of provider education, data collection and visualization tool refinement, we performed a prospective implementation of the protocol over a 6-month time frame (February 2023 to July 2023). Daily review of patients that met inclusion criteria was conducted, protocol compliance was validated against the automated system evaluation, and providers were contacted individually to discuss discrepancies and reinforce the protocol.

Monthly, stakeholders from the units participating in the study and unit leadership were provided with results of the protocol’s implementation and progressive safety outcomes. The goal for this phase was to assess appropriate diagnosis of pneumonia based on the defined criteria, decrease the rate of inappropriate fungal and mycobacterial cultures, and decrease the average duration of antibiotics.

### 2.4. Statistical Analysis

Patient characteristics are summarized using medians and interquartile ranges (IQRs) for continuous variables and counts and percentages for categorical variables. These characteristics are presented separately for patients who were treated pre-protocol implementation versus post-protocol.

To satisfy distributional assumptions, cumulative spectrum score, duration of antibiotics, hospital length of stay, ICU length of stay, and ICU mechanical ventilation days were analyzed using log transformation. Continuous outcomes were analyzed using univariate linear regression and binary outcomes were analyzed using univariate logistic regression. In all cases, the explanatory variable of interest was treatment pre- or post-protocol. Due to the limited sample size, we did not conduct covariate-adjusted analyses.

Complete case analysis was used to handle missing data, which were limited. Two-tailed *p*-values of 0.05 or less were considered statistically significant. Data management and statistical analysis were performed in SAS version 9.4 (SAS Institute Inc., Cary, NC, USA).

## 3. Results

### 3.1. Phase 1: Chart Review for Protocol Validation

From July to December 2022, 42 patients with antibiotics prescribed for an indication of respiratory tract infection were screened and 35 were included. Patients had a median age of 63 years (IQR 49–72) and were primarily admitted for elective CVS (60%). For the assessment of accurate diagnosis relative to likelihood of pneumonia, 51% of patients met the criteria for probable HAP/VAP, 9% for possible HAP/VAP, and 40% for unlikely HAP/VAP. Further demographic information can be found in Table 2.

From a subjective and objective marker perspective, patient criteria to meet pneumonia diagnosis were driven by hypoxia (86%), new lung consolidation on imaging (59%), and new or worsened leukocytosis (59%). Cultures were sent for 80% of patients, with 32% having positive results. No patient had a history of multidrug-resistant organisms (e.g., extended-spectrum beta-lactamase- or carbapenemase-producing organisms). Additional laboratory and physiologic data can be found in Table 3.

In this pre-protocol phase, only 23% of patients had appropriate diagnostic testing sent. Inappropriate treatment was driven by inappropriate duration of therapy. These and other outcomes are available in Table 4.

### 3.2. Phase 3: Prospective Implementation and Daily Audit

From February to July 2023, 46 patients with antibiotics prescribed for an indication of respiratory tract infection were screened and 39 were included. Patients had a median age of 66 years (IQR 57–73) and were primarily admitted for elective (38%) or emergent (21%) CVS. For the assessment of accurate diagnosis relative to likelihood of pneumonia, 77% of patients met the criteria for probable HAP/VAP, 5% for possible HAP/VAP, and 18% for unlikely HAP/VAP (Table 2).

Patients in the post-protocol group had a similar set of criteria to meet pneumonia diagnosis, with 64% experiencing hypoxia, new consolidation on imaging in 71%, and new or worsened leukocytosis in 64%, while 18% of patients had a history of multidrug-resistant organisms (Table 3).

Treatment protocol failure in post-protocol implementation was driven by inappropriate duration of therapy, primarily related to providers extending treatment beyond 3–5 days for culture-negative HAP/VAP syndromes (Table 4).

### 3.3. Comparative Outcomes

Duration of antibiotic treatment was not significantly reduced in the post-protocol group (6 days [IQR = 5.0, 10.0] vs. 7 days [IQR = 6.0, 9.0] pre-protocol, *p* = 0.36). Appropriate diagnostic testing improved significantly to 54% post-protocol vs. 23% in the pre-protocol group (*p* = 0.008). Though we observed a numeric decrease in patients unlikely to have HAP/VAP diagnoses in the post- vs. pre-protocol groups (18% vs. 40%), we did not find statistical significance. None of post- vs. pre-protocol hospital length of stay (26.2 days [16.7, 43.3] vs. 36.5 days [16.4, 53.2], *p* = 0.08), ICU length of stay (22.6 days [9.9, 30.4] vs. 11.3 days [6.5, 37.9], *p* = 0.73), or mechanical ventilation (9.1 days [2.4, 20.3] vs. 6.5 days [0.4, 19.2], *p* = 0.20) was significantly different between the groups. Median cumulative spectrum scores did not differ between groups either (56 pre-protocol [40, 64] vs. 49 [35, 70] post-protocol, *p* = 0.84) [16].

### 3.4. Automated Dashboard Assessment

To validate the accuracy of the rule-based logic, we compared the results from the clinician’s manual assignment of adherence to the protocols to the outcome returned by the automation. We observed that in 94.8% of all prospective cases, the automation assigned the same result as the clinician’s manual assignment of adherence or nonadherence to the protocols. There were two cases where the automation had assigned nonadherence to the diagnostic protocol due to unnecessary cultures, but the clinician’s manual assignment was that they had followed the adherence protocol. Upon retrospective review by the study team, it was determined that the automated assignment was correct based on the protocol.

When comparing the automated assigned result to the clinician’s manual assignment of treatment protocol adherence, we observed that in 74.3% of all prospective cases, the assignment of adherence was the same. In eight cases, the automation had assigned appropriate adherence to treatment protocol due to duration of therapy, where the clinician’s manual assignment had been adherence to treatment protocol. In two cases, the automation assigned appropriate duration of therapy where the clinician assigned non-adherence. On retrospective review by the study team, the automation had correctly assigned the appropriate adherence based on the established protocol for all cases.

## 4. Discussion

The ATS and IDSA guidelines for HAP and VAP syndromes combine objective and subjective criteria, namely, “new lung infiltrate plus clinical evidence that the infiltrate is of an infectious origin, with new onset of fever, purulent sputum, leukocytosis, and decline in oxygenation” [2]. While some of these objective criteria are not difficult to ascertain, subjective criteria make the diagnosis challenging. Further confusion can be added for clinicians by the infection prevention and control surveillance definitions of ventilator-associated events (VAEs) and ventilator-associated conditions defined by the National Health Care Safety Network (NHSN). This makes documentation of incidence rates of these syndromes in the hospital challenging [17]. In this study, after a baseline assessment of HAP/VAP diagnosis and management in the CVS-ICU, we aimed to clarify clinical diagnostic challenges and guide antimicrobial selection and duration through a diagnostic and treatment protocol. We saw a statistically significant improvement in appropriate culture testing. We additionally saw a numeric decrease in patients given antibiotics who did not meet the criteria for probable or possible HAP/VAP after protocol implementation.

Our protocols were developed and validated with the input of a variety of stakeholders, which aided in general support and uptake. We made the protocols deliberately “strict” to aim at the highest possible improvement in the clinical management of these syndromes. Colleagues in the CVS-ICU were aware of this protocol’s deliberately strict nature.

When comparing the pre- and post-protocol groups, we observed that a numerically larger percentage of patients post-implementation given antibiotics were considered to have probable HAP/VAP and a smaller percentage were given antibiotics with a classification of unlikely HAP/VAP (overall pneumonia comparison nonsignificant with a *p* value of 0.263). In the current landscape where ICU providers significantly over-diagnose nosocomial pneumonia, we hypothesize that the clear diagnostic criteria algorithm providers had to follow contributed to this numeric shift in patients receiving antibiotics who did not meet criteria for initiation [5]. The finding that only 80% of patients in the pre-protocol and 85% in the post-protocol group had cultures sent points to another challenge: patients who were not producing sputum or secretions in sufficient quantities were not able to have cultures obtained. This calls into question whether these patients had pneumonia at all, which further reinforces the importance of a protocolized approach to management. Cultures were sent based on the discretion of the primary intensive care unit teams taking care of the patient. If cultures were not sent, often empirical therapy was still given by the primary teams based on their clinical concern for pneumonia. This is a certain limitation of our post-protocol data analysis and could explain the lack of difference in antimicrobial therapy between groups. This also reflects the real-world scenario where antimicrobial clinical care in the ICU is often directed by primary ICU teams and not infectious disease teams. Empirical 5–7 day duration of therapy was even given on some occasions when cultures were negative owing to ICU provider concern for pneumonia based on variables such as purulent secretions on airway examination and perceived response to antimicrobials after weighing the risks vs. benefits of same.

We did also note a significant increase in the proportion of patients who had appropriate cultures collected. The previously described order set changes did not go live until several months into the protocol implementation, which contributed to what we perceived as a lower-than-expected uptake of culture sending. Even with lower-than-expected uptake, we estimate having saved 216 days (about 7 months) of unnecessary fungal culture retention and 378 days (about 1 year) of unnecessary mycobacterial culture retention. Empirical antimicrobial selection improved and the overall spectrum of antimicrobial use reduced after implementation of the protocol, though this did not reach statistical significance. We do believe these are nevertheless important gains, because avoiding unnecessary fungal and mycobacterial cultures has a significant advantage of preventing unnecessary antifungal and anti-mycobacterial therapeutic use. Improving empirical antimicrobial selection to institution-preferred cefepime as primary Gram-negative coverage and reducing the spectrum of antimicrobial use when cultures do not demonstrate *Pseudomonas* spp. or another organism requiring broad therapy also have multiple obvious benefits which are hard to demonstrate statistically.

Barriers to stewardship efforts in the ICU setting are well described and include provider diagnostic uncertainty, concern for lack of coverage of primary pathogen, and minimization of antibiotic toxicity [17,18]. Pickens et al. described ideal elements of antimicrobial stewardship in the ICU, which include multidisciplinary and multispecialty leadership, prospective audit and feedback, rapid diagnostics, clinical pathways, and computerized decision support, among others [18]. We sought to quantify a challenging clinical scenario in the CVS-ICU and work with local stakeholders to devise a protocol to improve provider diagnostic certainty with suspected pneumonia, but allow for rapid de-escalation if cultures remained negative and clinical status underwent rapid improvement.

In summary, we saw a reduction in inappropriate diagnostic testing and nonsignificant trends in reduction in mean duration of antimicrobial therapy for a nosocomial pneumonia episode and in the spectrum of antimicrobial exposure in our prospective patient population. This was without a statistically significant impact on outcomes including ICU length of stay, hospital length of stay, or time requiring invasive mechanical ventilation. Our next steps are to continue to build the automated process to minimize the daily man-hours needed for implementation with a goal to provide ongoing active dashboard availability to the ICU providers for self-monitored diagnosis and prescribing patterns. We additionally will continue to educate and promote the cessation of antibiotics in patients who have no growth from adequately sampled cultures.

Our study has several limitations. First, the logistical support needed for implementation of this project and protocol with the required input from various stakeholders makes external implementation of it challenging. Second, significant practice differences exist in managing these syndromes amongst providers in the CVS-ICU with regard to certain respiratory settings, etc., that certain clinicians may prefer to work with: for example, the standard approach in this unit for positive end-expiratory pressure on the ventilator is 10 mmHg, which in several studies is above the threshold to consider a diagnosis of VAP [15,17,19]. Third, the small sample limits our ability to identify statistically significant changes attributable to protocolized efforts. Lastly, ours is a part-retrospective and part-prospective project and hence has the inherent biases of these designs.

Our study, however, demonstrates that with good multidisciplinary stakeholder engagement, collaboration between IPAC and antimicrobial stewardship, utilization of a validated, rule-based logic to automate assessments and visual strategies, and management of difficult-to-diagnose and -treat syndromes in challenging and vulnerable patients can be improved. A common challenge for clinicians managing these patients on the frontline in the ICU is clinical complexity and the fragile nature of the patient’s clinical status. However, with application of a well-designed strategy with collaboration, review, and feedback, it is possible to make gains in IPAC and antimicrobial stewardship targets, even in a complex critically ill patient population.

## 5. Conclusions

Critically ill patients in the intensive care unit, particularly those in the CVS-ICU setting, present with multiple etiologies of inflammatory syndromes, and diagnostic certainty for pneumonia is extremely challenging. This patient population is difficult to manage due to complex care and clinical confounders regarding comorbidities. Antimicrobial management and the impact of diagnostic testing in these patients is often an afterthought. Through this project, we instituted a multidisciplinary approach for achieving improvement in diagnosis and management of this syndrome, including downstream improvements in antimicrobial and diagnostic stewardship and infection prevention and control.

## Figures and Tables

**Table 1:** Patient-specific factors assessed to measure likelihood of nosocomial pneumonias and appropriate treatment course.

Clinical Presentation Factors	Objective Factors
Increased or purulent secretions	New PEEP > 10 in intubated patient
New lung consolidation on chest imaging	Respiratory rate > 25 bpm in non-intubated patient
Presence of fluid overload or cardiogenic shock	New FiO_2_ > 0.4
Stability on day 3 and day 5	Fever (temperature ≥ 38.2 °C)
	New leukocytosis (WBC > 12)
	History of MDROs cultured in prior 90 days
	Duration of antibiotic therapy
	Cultures ordered and their results

PEEP: positive end-expiratory pressure; bpm: breaths per minute; FiO_2_: fraction of inspired oxygen; WBC: white blood cell; MDROs: multidrug-resistant organisms.

**Table 2:** Patient characteristics, *n* = 74.

	Pre-Protocol (*n* = 35)	Post-Protocol (*n* = 39)
Age, median (IQR)	63.0 (49.0, 72.0)	66.0 (57.0, 73.0)
Sex, *n* (%)		
Female	13 (37%)	13 (33%)
Male	22 (63%)	26 (67%)
Type of hospitalization, *n* (%), N = 69		
Elective	21 (60%)	13 (38%)
Emergency	7 (20%)	7 (21%)
Trauma	0 (0%)	1 (3%)
Urgent	7 (20%)	0 (0%)
SOFA score (at 24 h), median (IQR)	12.0 (9.0, 14.0)	12.0 (5.0, 14.0)
APACHE3 score (at 24 h), median (IQR)	124.0 (80.0, 144.0)	105.0 (66.0, 125.0)
Accurate diagnosis		
Probable HAP/VAP	18 (51%)	30 (77%)
Possible HAP/VAP	3 (9%)	2 (5%)
Unlikely HAP/VAP	14 (40%)	7 (18%)

QR: interquartile range; SOFA: sequential organ failure assessment; APACHE: acute physiology and chronic health evaluation; HAP: hospital-acquired pneumonia; VAP: ventilator-associated pneumonia.

**Table 3:** Laboratory and physiologic data.

	Pre-Protocol (*n* = 35)	Post-Protocol (*n* = 39)
PEEP, median (IQR), N = 56, mmHg	8.0 (8.0, 10.0)	10.0 (8.0, 13.0)
Tachypnea, *n* (%),	24 (69%)	24 (62%)
Hypoxia, *n* (%)	30 (86%)	25 (64%)
FiO_2_, median (IQR), N = 66	47.5 (40.0, 60.0)	45.0 (40.0, 60.0)
Temperature (°C), median (IQR), degrees C	36.8 (36.6, 37.4)	37.5 (37.2, 38.2)
New lung consolidation, *n* (%) N = 72	17 (50%)	27 (71%)
WBC, median (IQR), cells ×10^9^/L	15.0 (12.2, 22.2)	15.6 (11.4, 19.1)
New or worsened leukocytosis, *n* (%), N = 73	20 (59%)	25 (64%)
Fluid overloaded or Cardiogenic shock, *n* (%)	13 (37%)	13 (33%)
Culture sent, *n* (%)	28 (80%)	33 (85%)
Positive culture, *n* (%), N = 61	9 (32%)	18 (55%)
Symptoms, *n* (%)	29 (83%)	35 (90%)
History of multidrug-resistant organisms, *n* (%)	0 (0%)	7 (18%)

PEEP: positive end-expiratory pressure; IQR: interquartile range; FiO_2_: fraction of inspired oxygen; WBC: white blood cell.

**Table 4:** The impact of protocol implementation on primary and secondary outcomes.

			Unadjusted		
	Pre-Protocol (*n* = 35)	Post-Protocol (*n* = 39)	Estimate *	95% CI	*p*-Value
Primary					
Nosocomial pneumonia, *n* (%)	27 (77%)	34 (87%)	1.42	(0.77, 2.62)	0.263
Appropriate diagnostic testing, *n* (%)	8 (23%)	21 (54%)	1.98	(1.20, 3.29)	0.008
Appropriate treatment, *n* (%)	21 (60%)	19 (49%)	0.80	(0.50, 1.26)	0.235
Reason for treatment protocol nonadherence *n* (%), N = 34			-	-	-
Inappropriate duration of therapy, *n* (%)	14 (100%)	19 (95%)			
Inappropriate antibiotic and duration of therapy	0 (0%)	1 (5%)			
Secondary appropriate treatment, *n* (%)	22 (63%)	26 (67%)	1.09	(0.67, 1.75)	0.732
Cumulative spectrum score, median (IQR) a *a*	56.0 (40.0, 64.0)	49.0 (35.0, 70.0)	0.97	(0.76, 1.25)	0.842
Secondary					
Duration of antibiotics, days, median (IQR) a *a*	7.0 (6.0, 9.0)	6.0 (5.0, 10.0)	0.90	(0.71, 1.13)	0.356
Hospital length of stay, days, median (IQR) a *a*	36.5 (16.4, 53.2)	26.2 (16.7, 43.3)	0.72	(0.50, 1.04)	0.084
ICU length of stay, days, median (IQR) a *a*	11.3 (6.5, 37.9)	22.6 (9.9, 30.4)	1.25	(0.83, 1.89)	0.283
ICU mechanical ventilation days, median (IQR) a *a*	6.5 (0.4, 19.2)	9.1 (2.4, 20.3)	1.70	(0.77, 3.76)	0.197

Reference group is pre-protocol. IQR: interquartile range. * Estimates are odds ratios for nosocomial pneumonia, accurate diagnosis, appropriate diagnostic testing, appropriate treatment, and appropriate secondary treatment. Estimates are the multiplicative change in the outcome for cumulative spectrum score, duration of antibiotics, hospital and ICU length of stay, and ICU mechanical ventilation days. a *a* Data were log-transformed for normality.

## Data Availability

The original contributions presented in the study are included in the article, further inquiries can be directed to the corresponding author/s.
